# Maternal food allergy is associated with daughters’ menarche in early adolescence

**DOI:** 10.1186/s13223-019-0371-0

**Published:** 2019-09-11

**Authors:** Jennifer Lisa Penner Protudjer, Elissa Michelle Abrams, Anita Luba Kozyrskyj, Allan Barry Becker

**Affiliations:** 1 Department of Pediatrics and Child Health, The University of Manitoba, 753 McDermot Avenue, Winnipeg, MB R3E 0T6 Canada; 2 George and Fay Yee Centre for Healthcare Innovation, Winnipeg, Canada; 3 The Children’s Health Research Institute of Manitoba, Winnipeg, Canada; 4 Institute of Environmental Medicine, Karolinska Institutet, Stockholm, Sweden; 5 Department of Food and Human Nutritional Sciences, University of Manitoba, Winnipeg, Canada; 6 Department of Pediatrics, The University of Alberta, Edmonton, Canada

**Keywords:** Epidemiology, Females, Food allergy, Maternal, Menarche

## Abstract

**Rationale:**

Associations between allergic disease and puberty amongst females have been widely studied. However, this association has received less attention in multigenerational populations. To this end, we sought to examine maternal allergic disease status ever, and daughters’ menarche.

**Methods:**

In a cohort of children born in 1995, in Manitoba, Canada, we considered maternal allergic disease ever to daughters’ age 7–8 years, and daughters’ menarche at ages 12–14 years. We included all participants for whom we had information on both the exposure and the outcome of those eligible. Data were analysed using descriptive statistics and logistic regression, with adjustment for confounding variables.

**Results:**

Overall, the prevalences of maternal allergic diseases were 28.6% for asthma 18.8% for food allergy, 27.3% for eczema and 45.5% for rhinitis. By age 12–14 years, 41.6% (64/159) girls had reached menarche. Maternal food allergy was significantly associated with daughters’ menarche (OR 4.39, 95% CI 1.51–12.73), whereas no association was found for maternal asthma, eczema or rhinitis. With consideration to comorbid disease, a combination of maternal asthma + food allergy was associated with daughters’ menarche by age 12–14 years (OR 6.41; 95% CI 1.32–31.01).

**Conclusions:**

Maternal food allergy ever is associated with daughters’ menarche by age 12–14 years.

A gender switch in allergic disease has been noted during the pubertal years [1]. Less is known about the effect of allergic disease on timing of pubertal development. In one Swedish study, no clear associations were found between asthma, including timing of onset and phenotypes, and pubertal staging [2]. To our knowledge, no studies have examined maternal allergic disease and the timing of daughters’ menarche.

To investigate this knowledge gap, we used data from 154 mother-daughter dyads from the Study of Allergy, Genes and the Environment (SAGE) [3], a general population-based cohort of children at high- and low risk for asthma. In this exploratory analysis, data on maternal allergic disease (self-reported asthma, food allergy, eczema and/or rhinitis) were collected several years prior to daughters’ menarche. Thus, this study design provided the ability to estimate the impact of maternal allergic disease and daughters’ subsequent age of menarche. As maternal stress is associated with early pubertal onset for their daughters [4], we also considered maternal depression shortly after their daughters’ births in our analyses. Our primary aim was to examine the association between individual maternal allergic diseases and daughters’ menarche. Our secondary aim was to consider the timing of disease onset and disease comorbidities, and daughters’ menarche.

Briefly, in 2002, 723 children born in Manitoba, Canada in 1995 were recruited to SAGE. Children and their families participated in al assessment and completed questionnaires when the children were ages 7–9 years (baseline), ages 10–11 (late childhood) and 12–14 years (adolescence; 68% retention). At baseline, mothers reported if they had experienced any previous symptoms of asthma, food allergy, eczema or rhinitis. Asthma was further dichotomised as childhood onset (≤ 12 years) vs. post-pubertal onset (13+ years). At the adolescent visit, daughters self-reported breast development, per Tanner staging [5], which we categorised as early vs. middle/late, whereas mothers reported if their daughters had reached menarche (no vs. yes).

At baseline, mothers provided information on breastfeeding, birthweight, gestational age, and maternal smoking and education. Additionally, mothers reported household income, which we dichotomised at $50,000, to approximately align with the median Manitoba income at baseline. In late childhood, mothers reported whether they felt depressed or hopeless following their daughter’s birth in 1995. Possible answers to this question were dichotomised as no vs. yes.

In adolescence, girls’ heights and weights were measured in triplicate by research staff. The mean measures were taken, from which body mass index (BMI) was calculated. As hip and waist measures (in centimeters), converted to waist-hip ratio, did not substantially alter point estimates (< 0.10) or change statistical significance compared to analyses in which BMI was considered, we present only the results in which BMI was considered.

Data were described using n, %, mean, and 95% confidence intervals (95% CI). Analytic statistics included logistic regression, reported as odds ratios (OR) and corresponding 95% CI. Potential confounding variables were identified using directed acyclic graphs [4], and considered in partially and fully adjusted models. Statistical significance was set at p < 0.05. Data were analysed using Stata 13.1 (College Station, TX). Ethical permission was granted by the University of Manitoba Health Research Ethics Board (HS14742(HS2002:078)).

Of the 470 participants seen in adolescence, 203 were girls, for whom menarche data were available for 154 (75.9%) This constituted our study population. Mothers reported predominantly Caucasian ethnicity, and the majority had post-secondary education and had breastfed their daughters (Table 1). Approximately 30% (44/154) of mothers had asthma, of whom 38.4% (17/44) had pre-pubertal asthma. Other allergic diseases were also common.

**Table 1:** Demographic characteristics of mother-daughter dyads (N = 154)

	n	%
Maternal characteristics		
Ever smoked	66	42.9
Ethnicity		
Caucasian	133	86.4
Indigenous	15	9.7
Visible minority	6	3.9
Highest education		
No post-secondary	19	13.2
Post-secondary	125	86.8
Income ($)		
< 49,999	46	32.2
> 50,000	97	67.8
Region		
Urban	90	58.4
Rural	64	41.6
Ever breastfed daughter	130	84.4
Maternal depression after daughter’s birth		
No	74	56.1
Yes	58	43.9
Allergic disease		
Asthma		
Ever	44	28.6
Pre-pubertal	18	42.9
Food allergy	29	18.8
Eczema	42	27.3
Rhinitis	70	45.5
Daughters' characteristics		
Born at 38+ weeks	136	88.3
Thelarche^a^	109	79.5
Menarche^b^	64	41.6

	Mean ± SD	
Birthweight (kg)	3.35 ± 0.60	
Gestational age (weeks)	39.6 ± 1.7	
BMI in adolescence	21.0 ± 5.2	

^a^Middle/late thelarche, per daughters’ reported Tanner staging

^b^Based on daughters’ reports

No associations were found between maternal allergic disease and daughters’ thelarche (Table 2). In contrast, in unadjusted and partially adjusted models, maternal asthma trended towards an association with daughters’ menarche by the adolescent visit, whereas this association was significant for maternal food allergy (Table 2). In models adjusted for all covariates except maternal depression, the statistically significant association between maternal food allergy and daughters’ menarche persisted (OR 3.02; 95% CI 1.15–7.93; p < 0.03). In contrast, neither maternal eczema nor rhinitis were associated with daughters’ menarche. Adding the covariate, maternal depression, insubstantially altered the corresponding point estimates, thereby further strengthening the results. The difference in findings between thelarche and menarche may be partly attributable to differences in reporting (daughter vs. mother, respectively), as reflected by a moderate correlation between these variables (*r* 0.498).

**Table 2:** Associations between maternal allergic disease and daughters’ thelarche and menarche by age 12–14 years

	Unadjusted		Model 1^a^		Model 2^b^	
	OR	95% CI	OR	95% CI	OR	95% CI
II: Thelarche (N = 137 dyads)						
Asthma	0.72	0.30; 1.72	0.58	0.21; 1.63	0.47	0.16; 1.40
Food allergy	1.69	0.54; 5.35	1.52	0.44; 5.17	1.88	0.48; 7.38
Eczema	1.30	0.50; 3.36	1.25	0.43; 3.69	1.18	0.39; 3.60
Rhinitis	1.01	0.44; 2.33	0.62	0.23, 1.68	0.68	0.24; 1.96
III: Menarche (N = 154 dyads)						
Asthma	1.84	0.91; 3.74	1.52	0.64; 3.64	1.41	0.53; 3.71
Food allergy	2.35	1.03; 5.36	3.02	1.15; 7.93	4.39	1.51; 12.73
Eczema	0.94	0.46; 1.93	1.03	0.43; 2.48	1.45	0.57; 3.70
Rhinitis	1.10	0.58; 2.10	0.92	0.41; 2.05	0.97	0.39; 2.42

^a^Adjusted for daughter’s birthweight and household income, maternal smoking and daughters’ BMI in adolescence

^b^Adjusted for daughter’s birthweight, household income, maternal smoking, daughter’s BMI in adolescence, and maternal depression following daughter’s birth

Given the null findings between maternal allergic disease and daughters’ thelarche, we performed no further similar analyses. However, we did consider timing of maternal asthma onset and one vs. both of these allergic diseases, in association with daughters’ menarche. Nearly all (95.5%; 42/44) mothers with asthma reported the age at which they had their first asthma exacerbation. No associations were found between pre- vs post-pubertal first maternal asthma exacerbation and daughters’ menarche (Table 3). Similarly, no statistically significant associations were found between maternal asthma or food allergy ever and daughters’ menarche in partially adjusted and fully adjusted models. Both maternal asthma and food allergy increased the odds of daughters’ menarche more than five-fold (fully adjusted: OR 5.71; 95% CI 1.20–27.3). Although the numbers for some sub-analyses were small, the point estimates were similar to those from analyses of the entire study population, indicating a robust association. Moreover, these analyses were robust to adjustment for BMI, which is also associated with early puberty [5]. Data on maternal allergic disease were based on self-report, not clinical testing. However, by using maternal data from baseline and reports of daughters’ pubertal development at later ages, we eliminate any potential reporting bias. We acknowledge that out outcome, daughters’ menarche, was reported by mothers rather than the girls themselves. However, any differences in classification of menarche are likely to be non-differential. In addition, we were unable to consider maternal age at menarche, as these data were not collected in our study.

**Table 3:** Associations between maternal asthma, including timing of onset, and history of food allergy, and daughters' menarche by age 12–14 years (N = 154 dyads)

	Unadjusted		Model 1^a^		Model 2^b^	
	OR	95% CI	OR	95% CI	OR	95% CI
First maternal asthma exacerbation						
Prepubertal	1.00		1.00		1.00	
Post-pubertal	0.54	0.16; 1.86	0.53	0.10; 2.90	0.34	0.05; 2.34
Maternal asthma						
None	1.00		1.00		1.00	
First maternal asthma exacerbation before puberty	2.64	0.95; 7.36	2.11	0.59; 7.55	2.73	0.73; 10.19
First maternal asthma exacerbation after puberty	1.42	0.58; 3.47	1.15	0.38; 3.52	0.92	0.84; 8.70
Maternal comorbid disease						
No asthma or food allergy	1.00		1.00		1.00	
Asthma or food allergy	*2.07*	*1.04; 4.12*	1.71	0.72; 4.07	1.71	0.64; 4.59
Both asthma and food allergy	4.00	0.93; 17.1	*4.53*	*1.00; 20.6*	*6.41*	*1.32; 31.01*

Statistically significant values are in italic

^a^Adjusted for daughter’s birthweight household income, maternal smoking and daughter’s BMI in adolescence

^b^Adjusted for daughter’s birthweight, household income, maternal smoking, and daughter’s BMI in adolescence and maternal depression following daughter’s birth

To our knowledge, this is the first study on maternal food allergy and daughters’ pubertal development, and highlights the need to consider the impact of maternal allergic disease using multigenerational studies. Although there are diverging results as to which parent’s allergic disease status confers greater risk [6, 7], a greater maternal impact may be attributable to their role as the parent of origin, as well as environmental and immunological interactions with the offspring during pregnancy and birth. Accordingly, we restricted our analyses to mother-daughter dyads.

There is substantial, but collectively inconclusive evidence surrounding allergic disease and puberty [6, 8], asthma and subsequent menarche [7, 9]. Whereas biological plausibility has been described for this association in a single generation, it remains unclear why maternal food allergy is associated with daughters’ menarche in early adolescence. Maternal atopic disease, especially food allergy, may well indicate the start of a multigenerational cascade of chronic, inflammatory disease. As such, this observation warrants further investigation as early menarche increase the daughters’ risk of other chronic conditions, including type 2 diabetes [10] and cardiovascular disease [11]. Likewise, physicians treating girls whose mothers have food allergy may wish to be mindful of early menarche, and carefully monitor factors, such as body weight and blood glucose, which increase the risk of cardiovascular disease.

In conclusion, our study demonstrates an association between maternal food allergy alone, or in combination with asthma, and daughters’ menarche in early adolescence.

## Data Availability

The datasets analysed in the current study are not publicly available due to the sensitive nature of data collected from minor children.

## Abbreviations

BMI: body mass index
OR: odds ratio
SAGE: study of allergy, genes and the environment
95% CI: 95th percent confidence interval

## References

[1] Almqvist C, Worm M, Leynaert B, working group of GA2LEN WP 2.5 Gender (2008). Impact of gender on asthma in childhood and adolescence: a GA2LEN review. Allergy.

[2] Protudjer JLP, Lundholm C, Bergström A, Kull I, Almqvist C (2015). The influence of childhood asthma on puberty and height in Swedish adolescents. Pediatr Allergy Immunol.

[3] Kozyrskyj A, HayGlass K, Sandford A, Pare P, Chan-Yeung M, Becker A (2009). A novel study design to investigate the early-life origins of asthma in children (SAGE study). Allergy.

[4] Shrier I, Platt RW (2008). Reducing bias through directed acyclic graphs. BMC Med Res Methodol.

[5] Lazzeri G, Tosti C, Pammolli A, Troiano G, Vieno A, Canale N (2018). Overweight and lower age at menarche: evidence from the Italian HBSC cross-sectional survey. BMC Womens Health.

[6] Keller T, Hohmann C, Standl M, Wijga AH, Gehring U, Melen E (2018). The sex-shift in single disease and multimorbid asthma and rhinitis during puberty - a study by MeDALL. Allergy.

[7] Protudjer JL, Lundholm C, Bergstrom A, Kull I, Almqvist C (2015). The influence of childhood asthma on puberty and height in Swedish adolescents. Pediatr Allergy Immunol.

[8] Shah R, Newcomb DC (2018). Sex bias in asthma prevalence and pathogenesis. Front Immunol.

[9] Protudjer JL, Lundholm C, Almqvist C (2015). Asthma and height in twins: a cohort and within-pair analyses study. Twin Res Hum Genet.

[10] Janghorbani M, Mansourian M, Hosseini E (2014). Systematic review and meta-analysis of age at menarche and risk of type 2 diabetes. Acta Diabetol.

[11] Luijken J, van de Schouw YT, Mensink D, Onland-Moret NC (2017). Association between age at menarche and cardiovascular disease: a systematic review on risk and potential mechanisms. Maturitas.

